# A simple, randomized algorithm for diagonalizing normal matrices

**DOI:** 10.1007/s10092-025-00654-z

**Published:** 2025-07-25

**Authors:** Haoze He, Daniel Kressner

**Affiliations:** Institute of Mathematics, École Polytechnique Fédérale de Lausanne (EPFL), 1015, Lausanne, Switzerland

**Keywords:** Randomized numerical linear algebra, Eigenvalue problems, Normal matrices, Commuting matrices, 65F15, 15A27, 68W20

## Abstract

We present and analyze a simple numerical method that diagonalizes a complex normal matrix *A* by diagonalizing the Hermitian matrix obtained from a random linear combination of the Hermitian and skew-Hermitian parts of *A*.

## Introduction

A matrix $A \in {\mathbb {C}}^{n\times n}$ is called normal if $AA^* = A^* A$, where $A^*$ denotes the Hermitian transpose of *A*. Equivalently, the Hermitian and skew-Hermitian parts of *A* commute:

1 $$\begin{aligned} HS = SH, \quad H:= (A+A^*)/2, \quad S:= (A-A^*) /2. \end{aligned}$$

It is a basic linear algebra fact that *A* is normal if and only if it can be diagonalized by a unitary matrix *U*, that is, $U^* A U$ is diagonal.

Our recent work [12] on diagonalizing commuting matrices suggests a simple method for performing the diagonalization of a general normal matrix *A*: Draw two independent random numbers $\mu _H, \mu _S \in {\mathbb {R}}$ from the standard normal distribution $\mathcal N(0,1)$ and compute a unitary matrix *U* that diagonalizes the *Hermitian* matrix $\mu _H H + \mu _S \textrm{i} S$. This results in the following algorithm:
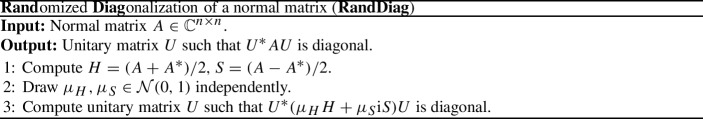


As we will explain in Sect. 2, RandDiag succeeds with probability one in exact arithmetic and remains fairly robust in the presence of errors introduced due to, e.g., roundoff. The main computational cost is in line 3, which directly benefits from decades of research and development on Hermitian eigenvalue solvers [4, 11, 18] and software, such as LAPACK [2]. This makes RandDiag very simple to implement in scientific computing software environments. For example, these are the Matlab and Python implementations of RandDiag:
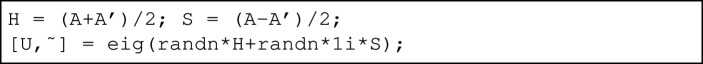

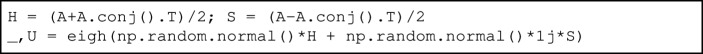


Compared to the Hermitian case, the case of a general normal matrix *A* is much less developed. In particular, we are not aware of a single publicly available implementation of an eigenvalue solver tailored to (complex) normal matrices, despite the fact that the development of such algorithms is a classical topic in numerical analysis. Already in 1959, Goldstine and Horwitz [10] proposed and analyzed a Jacobi-like method that annihilates the off-diagonal part of *A* by alternatingly applying Givens rotations. Ruhe [16] established local quadratic convergence of this method when applying Givens rotations in cyclic order and developed later on, in [17], a modified version for finding the normal matrix that is nearest (in Frobenius norm) to a given matrix. Since then, research on Jacobi methods for normal matrices has fallen nearly dormant, with the notable exception of [5]. However, two closely related problems have continued to attract interest: (1) Several algorithms have been developed for the more general task of simultaneously diagonalizing (nearly) commuting Hermitian matrices, including Jacobi-like methods [6, 7], optimization-based methods [21, 22], a “do-one-then-do-the-other” (DODO) approach [20], as well as randomized methods; see [12] and the references therein. By applying them to the commuting Hermitian matrices $H, \textrm{i}S$, any of these algorithms can be used to diagonalize a normal matrix $A = H + S$. Very recently [14], the DODO approach was adapted to block diagonalize a *real* normal matrix with a *real* orthogonal matrix. (2) When *A* is unitary, its Hessenberg form can be described by $\mathcal O(n)$ parameters, the so called Schur parameters, which is the basis of efficient algorithms [1, 3] for diagonalizing *A*. Unitary eigenvalue problems play an important role in mathematical physics; a recent application to modelling thermal conductivity can be found in [13]. The unitary variant of the QR algorithm described in [3] has been implemented in the eiscor Fortran 90 package.^1^

RandDiag is not only very simple but it is also fast, as demonstrated by our numerical experiments in Sect. 3. Experience [8] has shown that significant efforts are needed to make Jacobi-like methods, like the one by Goldstine and Horwitz, competitive. Even for the special case of a unitary matrix *A*, already the initial reduction to Hessenberg form required by the unitary QR algorithm can be more costly than RandDiag. Note, however, that not all applications may require this reduction; see [9] for an example.

## Analysis of RandDiag

We start our analysis of RandDiag by showing that it almost surely returns the correct result in the absence of error.

### Theorem 1

Given a normal matrix *A*, the unitary matrix *U* returned by RandDiag diagonalizes *A* with probability 1.

### Proof

The result follows from Theorem 2.2 in [12], which is concerned with the more general situation of diagonalizing a family of commuting matrices. For the convenience of the reader, we reproduce and simplify the argument for the situation at hand.

Because the matrices *H* and *S* defined in (1) commute, there exists a unitary matrix $U_0$ jointly diagonalizing *H* and $\mathrm i S$:

2 $$\begin{aligned} U_0^* H U_0 = \Lambda _H, \quad \mathrm U_0^* \textrm{i} S U_0 = \Lambda _S, \end{aligned}$$

with real diagonal matrices $\Lambda _H = \text {diag}( \lambda _1^{(H)},\ldots , \lambda _n^{(H)})$, $\Lambda _S = \text {diag}( \lambda _1^{(S)},\ldots , \lambda _n^{(S)})$. Clearly, this implies $U_0^* (\mu _H H + \mu _S \mathrm i S) U_0 = \mu _H \Lambda _H + \mu _S \Lambda _S.$ On the other hand, the matrix *U* computed by RandDiag also diagonalizes the same matrix: $U^*( \mu _H H + \mu _S \mathrm i S) = \widetilde{\Lambda }$. The diagonal matrix $\widetilde{\Lambda }$ contains the same eigenvalues as $\mu _H \Lambda _H + \mu _S \Lambda _S$, but they may appear in a different order on the diagonal. Hence, by reordering the diagonal entries of $\widetilde{\Lambda }$, there is a permutation matrix $\Pi$ such that

$$ \Pi ^* U^* (\mu _H H + \mu _S \mathrm i S) U \Pi = \Pi ^* \widetilde{\Lambda }\Pi = \mu _H \Lambda _H + \mu _S \Lambda _S. $$

Defining $V:= U_0^* U {\Pi }$, the two relations above imply

$$ V^* (\mu _H \Lambda _H + \mu _S \Lambda _S) V = \mu _H \Lambda _H + \mu _S \Lambda _S. $$

In other words, the unitary matrix *V* commutes with the diagonal matrix $\mu _H \Lambda _H + \mu _S \Lambda _S$, which implies that $v_{ij} = 0$ for any $i,j \in \{1,\ldots ,n\}$ such that

3 $$\begin{aligned} \mu _H \lambda _i^{(H)} + \mu _S \lambda _i^{(S)} \not = \mu _H \lambda _j^{(H)} + \mu _S \lambda _j^{(S)}. \end{aligned}$$

Now consider any *i*, *j* such that $\lambda _i^{(H)} \not = \lambda _j^{(H)}$ or $\lambda _i^{(S)} \not = \lambda _j^{(S)}$. Because $\mu _H, \mu _S$ are independent continuous random variables, the relation $\mu _H (\lambda _i^{(H)} - \lambda _j^{(H)}) + \mu _S (\lambda _i^{(S)} - \lambda _j^{(S)}) = 0$ is satisfied with probability zero; see, e.g., [12][Lemma 2.1]. Therefore, with probability 1, the following hold: The relation (3) and, thus, $v_{ij} = 0$ are satisfied for all such *i*, *j*. In turn, *V* commutes with $\Lambda _H$ and with $\Lambda _S$. Multiplying the two relations (2) from the left with $V^*$ and from the right with *V*, we get $U^* H U = \Lambda _H$ and $\mathrm U^* \textrm{i} S U = \Lambda _S$. In particular, *U* diagonalizes $A = H+S$. $\square$

The proof of Theorem 1 reveals why randomness is needed in RandDiag. Suppose that, instead of choosing $\mu _H,\mu _S$ randomly, we hard-coded a choice $\mu _H = \alpha$, $\mu _S = \beta$ for arbitrary fixed $\alpha , \beta \in \mathbb R$ in RandDiag. Then RandDiag would fail for the matrix

$$ A = U_0 \begin{bmatrix} \beta + \textrm{i} \alpha &  0 \\ 0 &  0 \end{bmatrix} U_0^*, $$

with some unitary matrix $U_0 \not = I$. In this case, $\mu _H H + \mu _S \mathrm i S = 0$ and $U=I$ would be a viable (and likely) output of RandDiag. But, clearly, $U = I$ does not diagonalize *A*.

When working in finite-precision arithmetic, the assumption of Theorem 1, that *A* is exactly normal, is not reasonable, unless *A* has a particularly simple structure, like Hermitian or diagonal. Already representing the entries of a, say, unitary matrix as floating point numbers introduces an error (on the order of the unit round-off *u*) that destroys normality. The Hermitian eigenvalue solver utilized in line 3 of RandDiag introduces additional error. If a backward stable method is used in line 3, one can conclude that RandDiag is executed for a slightly perturbed matrix $A+E$, with $\Vert E\Vert _F = \mathcal O( u \Vert A\Vert _F)$. However, it is unlikely that *E* preserves normality; the best one can reasonably hope for in that situation is that the transformation returned by RandDiag diagonalizes *A* up to an off-diagonal error proportional to $\Vert E\Vert _F$. In the following, we indeed establish such a robustness property by utilizing results on randomized joint diagonalization from [12]. Note that ${{\,\textrm{offdiag}\,}}(\cdot )$ refers to the off-diagonal part of a matrix, obtained by setting its diagonal entries to zero.

### Theorem 2

Consider $n\times n$ Hermitian matrices $A_1, A_2, \tilde{A}_1 = A_1 + E_1$, $\tilde{A}_2 = A_2 + E_2$ such that $A_1 A_2 = A_2 A_1$. Let $\tilde{U}$ be a unitary matrix that diagonalizes $\mu _1 \tilde{A}_1 + \mu _2 \tilde{A}_2$ for independent $\mu _1,\mu _2 \sim \mathcal N(0,1)$. Then, for any $R > 1$, the inequality

$$\begin{aligned} \big ( \big \Vert {{\,\textrm{offdiag}\,}}(\tilde{U}^T\tilde{A}_1\tilde{U})\big \Vert _F^2 + \big \Vert {{\,\textrm{offdiag}\,}}(\tilde{U}^T\tilde{A}_2\tilde{U})\big \Vert _F^2 \big )^{1/2} \le R \big ( \Vert E_1\Vert _F^2 + \Vert E_2\Vert _F^2 \big )^{1/2} \end{aligned}$$

holds with probability at least $1-\frac{12}{\sqrt{\pi }} \frac{n^{3.5}}{R-1}$.

### Proof

The result follows directly from Theorem 3.6 in [12] for $d=2$ matrices, after a straightforward extension from the real symmetric to the complex Hermitian case. In particular, it can be easily verified that the invariant subspace perturbation bound from Lemma 3.2 in [12], which plays a crucial role in the proof, continues to hold in the complex case. $\square$

### Corollary 3

(Robustness of RandDiag to error) Given a matrix $\tilde{A} = A + E \in {\mathbb {C}}^{n\times n}$, such that *A* is normal, let $\tilde{U}$ denote the unitary matrix returned by RandDiag when applied to $\tilde{A}$. Then, for any $R > 1$, the inequality

$$\begin{aligned} \Vert {{\,\textrm{offdiag}\,}}(\tilde{U}^*\tilde{A} \tilde{U})\Vert _F \le R \Vert E\Vert _F \end{aligned}$$

holds with probability at least $1 - \frac{12}{\sqrt{\pi }} \frac{n^{3.5}}{R-1}$.

### Proof

In analogy to the decomposition $A = H+S$ from (1), we decompose $\tilde{A} = \tilde{H} + \tilde{S}$ with

$$\begin{aligned} \tilde{H} = H + E_H, \quad \tilde{S} = S + E_S, \quad E_H = (E+E^*)/2, \quad E_S = (E-E^*)/2. \end{aligned}$$

Because Hermitian and skew-Hermitian matrices are orthogonal to each other (in the Frobenius inner product) and taking off-diagonal parts does not affect this property, it follows that

$$\begin{aligned} \Vert {{\,\textrm{offdiag}\,}}(\tilde{U}^*\tilde{A} \tilde{U})\Vert _F^2 =&\Vert {{\,\textrm{offdiag}\,}}(\tilde{U}^*\tilde{H} \tilde{U}) + {{\,\textrm{offdiag}\,}}(\tilde{U}^*\tilde{S} \tilde{U}) \Vert ^2_F\\ =&\Vert {{\,\textrm{offdiag}\,}}(\tilde{U}^*\tilde{H} \tilde{U}) \Vert _F^2 +\Vert {{\,\textrm{offdiag}\,}}(\tilde{U}^* \textrm{i} \tilde{S}\tilde{U})\Vert _F^2, \end{aligned}$$

as well as $\Vert E\Vert _F^2 = \Vert E_H\Vert _F^2 + \Vert \textrm{i} E_S\Vert _F^2$. The result of the corollary follows from Theorem 2 by setting $A_1 = H$, $A_2 = \textrm{i}S$, $E_1 = E_H$, and $E_2 = \textrm{i} E_S$. $\square$

The result of Corollary 3 guarantees, with high probability, an off-diagonal error proportional to the input error, if one allows for a magnification of the error by a factor that grows polynomially with *n*. Note that Corollary 3 also implies a backward error result because $\tilde{U}$ diagonalizes the normal matrix $\tilde{A} - \tilde{U}{{\,\textrm{offdiag}\,}}(\tilde{U}^*\tilde{A}\tilde{U})\tilde{U}^*$. In turn, this allows one to apply perturbation results, such as the Hoffmann-Wielandt theorem [19, Thm. IV.3.1], to draw conclusions on, e.g., the quality of the approximate eigenvalues obtained from the diagonal of $\tilde{U}^*\tilde{A} \tilde{U}$.

Note that, both, Theorem 2 and Corollay 3 make the (simplifying) assumption that the random variables involved are not affected by perturbations.

## Numerical experiments

In this section, we present a few numerical experiments to indicate the efficiency of RandDiag. With no (efficient) implementation of the Jacobi-like methods mentioned in Sect. 1 being available, the main competitor to RandDiag appears to be the (non-symmetric) QR algorithm [11] to compute the unitary matrix that transforms *A* into Schur form. Thanks to the backward stability of the QR algorithm, such an approach enjoys a strong robustness property in the sense of Corollary 3.

All experiments were carried out on a Dell XPS 13 2-In-1 with an Intel Core i7-1165G7 CPU and 16GB of RAM. We have implemented^2^ RandDiag in Python 3.8, calling LAPACK routines via Scipy for computing spectral and Schur decompositions. All experiments have been carried out in double precision and all execution times/errors are averaged over 100 runs with fixed input matrices. For the computed matrix $\tilde{U}$, we report the off-diagonal error $\Vert {{\,\textrm{offdiag}\,}}(\tilde{U}^*A \tilde{U})\Vert _F$.

### Synthetic data

We have tested RandDiag on random complex unitary matrices obtained from applying the QR factorization to $n\times n$ complex Gaussian random matrices. The obtained results are summarized in Tables 1, 2 and 3.

**Table 1:** Execution time and off-diagonal error for RandDiag vs. Schur decomposition applied to a random unitary matrix with $n=500$

	Time	Error mean	Error std	Error min	Error max
RandDiag	0.16s	$1.42\,\times \,10^{-10}$	$2.56\,\times \,10^{-10}$	$6.13\,\times \,10^{-12}$	$1.58\,\times \,10^{-09}$
Schur	0.64s	$2.58\,\times \,10^-{13}$	$5.05\,\times \,10^{-29}$	$2.58\,\times \,10^{-13}$	$2.58\,\times \,10^{-13}$

**Table 2:** Execution time and off-diagonal error for RandDiag vs. Schur decomposition applied to a random unitary matrix with $n=1000$

	Time	Error mean	Error std	Error min	Error max
RandDiag	0.58s	$7.88\,\times \,10^{-10}$	$3.60\,\times \,10^{-09}$	$2.54\,\times \,10^{-11}$	$3.54\,\times \,10^{-08}$
Schur	2.47s	$4.84\,\times \,10^{-13}$	0	$4.84\,\times \,10^{-13}$	$4.84\,\times \,10^{-13}$

**Table 3:** Execution time and off-diagonal error for RandDiag vs. Schur decomposition applied to a random unitary matrix with $n=1500$

	Time	Error mean	Error std	Error min	Error max
RandDiag	1.45s	$1.24\,\times \,10^{-09}$	$3.92\,\times \,10^{-09}$	$9.64\,\times \,10^{-11}$	$3.62\,\times \,10^{-08}$
Schur	6.50s	$6.72\,\times \,10^{-13}$	0	$6.72\,\times \,10^{-13}$	$6.72\,\times \,10^{-13}$

It turns out that RandDiag is up to four times faster than the standard Schur decomposition, at the expense of a few digits (on average three, at most five) of accuracy in the off-diagonal error. Note that this does not necessarily translate into reduced eigenvalue accuracy. Indeed, for the matrices from Tables 1, 2 and 3, extracting the eigenvalues from the diagonal of $U^* A U$ for the matrix *U* returned by RandDiag results in accuracy comparable to the Schur decomposition. In analogy to results for Hermitian matrices (see [15] and the references therein), the eigenvalue error appears to depend quadratically on the off-diagonal error for well separated eigenvalues.

To further analyze the accuracy of eigenvalues returned by RandDiag, we compute relative errors of the output eigenvalues for randomly generated normal matrices $A \in {\mathbb {C}}^{n \times n}$ of the form $U D U ^*$. The unitary matrix *U* is obtained as in the last experiment above, and *D* is diagonal with diagonal entries sampled from standard complex Gaussian. The relative errors are computed as follows. Let ${{\,\textrm{diag}\,}}(\cdot )$ denote the vector of diagonal entries of a matrix and $\tilde{U}$ be the output of RandDiag applied to *A*. Define $d_1 = {{\,\textrm{diag}\,}}(D) \in \mathbb {C}^n$ as the vector of original eigenvalues and $d_2 = {{\,\textrm{diag}\,}}(\tilde{U} ^* A \tilde{U}) \in \mathbb {C}^n$ as the vector of the output eigenvalues. The relative error is given by:

$$\Vert d_1 - P d_2\Vert _2 / \Vert d_1\Vert _2$$

where *P* is the the permutation matrix minimizing $\Vert d_1 - P d_2\Vert _2$. This optimal permutation is computed by solving a simple linear sum assignment problem, for which we leverage the implementation in Scipy.^3^ The results, summarized in Tables 4, 5, 6, show that RandDiag tends to be slightly more accurate than the Schur decomposition for this data, whereas the Schur decomposition yields more consistent results.

**Table 4:** Eigenvalue relative errors for RandDiag vs. Schur decomposition for a random normal matrix with $n=500$

	Error mean	Error std	Error min	Error max
RandDiag	$1.12\,\times \,10^{-15}$	$3.30\,\times \,10^{-17}$	$1.06\,\times \,10^{-15}$	$1.22\,\times \,10^{-15}$
Schur	$4.74\,\times \,10^{-15}$	$7.89\,\times \,10^{-31}$	$4.74\,\times \,10^{-15}$	$4.74\,\times \,10^{-15}$

**Table 5:** Eigenvalue relative errors for RandDiag vs. Schur decomposition for a random normal matrix with $n=1000$

	Error mean	Error std	Error min	Error max
RandDiag	$1.57\,\times \,10^{-15}$	$3.96\,\times \,10^{-17}$	$1.47\,\times \,10^{-15}$	$1.67\,\times \,10^{-15}$
Schur	$5.91\,\times \,10^{-15}$	$3.16\,\times \,10^{-30}$	$5.91\,\times \,10^{-15}$	$5.91\,\times \,10^{-15}$

**Table 6:** Eigenvalue relative errors for RandDiag vs. Schur decomposition for a random normal matrix with $n=1500$

	Error mean	Error std	Error min	Error max
RandDiag	$1.49\,\times \,10^{-15}$	$2.56\,\times \,10^{-17}$	$1.41\,\times \,10^{-15}$	$1.55\,\times \,10^{-15}$
Schur	$6.61\,\times \,10^{-15}$	$1.58\,\times \,10^{-30}$	$6.61\,\times \,10^{-15}$	$6.61\,\times \,10^{-15}$

For unitary matrices, using a structure-preserving QR algorithm [3] reduces the time needed to compute the Schur decomposition. However, it is unlikely that even a well-tuned implementation will significantly outperform RandDiag, because of the required initial reduction to Hessenberg form. For $n = 1000$, Hessenberg reduction requires 0.44*s* in Python. In Matlab, we found that Hessenberg reduction requires 0.31s but RandDiag only requires 0.21s.

**Fig. 1:**
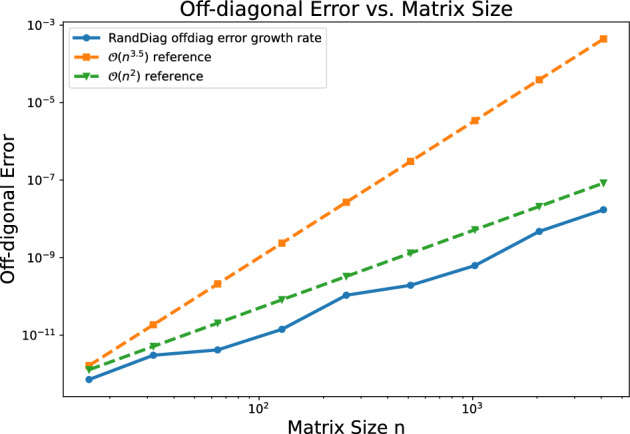
Error growth rate of RandDiag vs. matrix size on log-l-og scale

Next, we examine the growth of error with respect to matrix size for RandDiag. Figure 1 illustrates the average off-diagonal errors over 100 runs of randomly generated unitary matrices with sizes ranging from 8 to 4096, in powers of 2. Note that the plot uses a log-log scale. Empirically, the growth rate is closer to $\mathcal {O}(n^2)$, indicating that the $\mathcal {O}(n^{3.5})$ bound in Corollary 3 is too pessimistic. Tightening this $\mathcal {O}(n^{3.5})$ bound remains an open question for future research.

### Thermal conductivity estimation

A thermal conductivity model from [13] features unitary matrices of the form

$$\begin{aligned} U_F = U_{\text {int}}U_0, \quad U_0 = \bigotimes _{j=1}^{L}d_j, \quad U_{\text {int}} = \prod _{j=1}^{L-1} I_{2^{{\pi (j)}-1}} \otimes u_{\pi (j),\pi (j)+1} \otimes I_{2^{L-{\pi (j)}-1}} \end{aligned}$$

where $\otimes$ denotes the usual Kronecker product, *L* corresponds to the number of states, each $d_j$ is a (uniformly distributed) random $2 \times 2$ unitary matrix, and $u_{j,j+1} = \exp [\textrm{i} M_{j,j+1}]$ where $M_{j,j+1}$ is a random matrix drawn from the $4 \times 4$ Gaussian unitary ensemble (GUE) normalized such that $\mathbb {E}[\text {trace}(M^2)] = 2$. Note that $U_{\text {int}}$ describes the nearest neighbor interaction between states. The result of RandDiag applied to $U_F$ for $L = 11$ (that is, $n = 2^{11}=2048$) is shown in Table 7. This time, we observe a speedup by nearly a factor of 6, at the expense of more than 4 digits of accuracy in the off-diagonal error. The level of accuracy attained by RandDiag usually suffices for this kind of applications.

**Table 7:** Execution time and off-diagonal error for RandDiag vs. Schur decomposition applied to thermal conductivity model

	time	error
RandDiag	3.49s	$1.26\,\times \,10^{-09}$
Schur	20.73s	$\ 9.24\,\times \,10^{-13}$

## Data Availability

All the data is available at https://github.com/haoze12345/Diagonalizing-Normal-Matrices.

## Footnotes

1 See https://github.com/eiscor/eiscor.

2 Our implementation as well as all files needed to reproduce the experiments can be found at https://github.com/haoze12345/Diagonalizing-Normal-Matrices.

3 https://docs.scipy.org/doc/scipy/reference/generated/scipy.optimize.linear_sum_assignment.html.
